# Expression of Concern: Park et al. Isorhamnetin Induces Cell Cycle Arrest and Apoptosis Via Reactive Oxygen Species-Mediated AMP-Activated Protein Kinase Signaling Pathway Activation in Human Bladder Cancer Cells. *Cancers* 2019, *11*, 1494

**DOI:** 10.3390/cancers18142357

**Published:** 2026-07-22

**Authors:** Cancers Editorial Office

**Affiliations:** MDPI, St. Alban-Anlage 66, 4052 Basel, Switzerland; cancers@mdpi.com

With this notice, the Cancers Editorial Office alerts readers to concerns regarding this article [1].

Following publication of this review article, concerns were raised about the integrity of Figures 3, 4A, 6A and 7A. The authors were contacted and asked to provide clarification and supporting information. While the authors cooperated with the investigation, they were unable to provide sufficient supporting material to fully validate the figures, and the concerns could not be conclusively resolved.

The Editorial Office and Editorial Board assessed the matter and determined that the issues identified do not appear to substantially affect the overall conclusions of the article.

This Expression of Concern is therefore issued to inform readers of the unresolved concerns relating to the figures mentioned above. The Editorial Board has no further action planned at this time. However, should additional information become available, further action may be taken in accordance with MDPI’s policies if deemed necessary.
