# Supplementary figures and images for: Isorhamnetin Induces Cell Cycle Arrest and Apoptosis Via Reactive Oxygen Species-Mediated AMP-Activated Protein Kinase Signaling Pathway Activation in Human Bladder Cancer Cells
Source: Cancers (Basel). 2019 Oct 4;11(10):1494. doi: 10.3390/cancers11101494 (PMC6826535; doi:10.3390/cancers11101494)

Figure 3-A

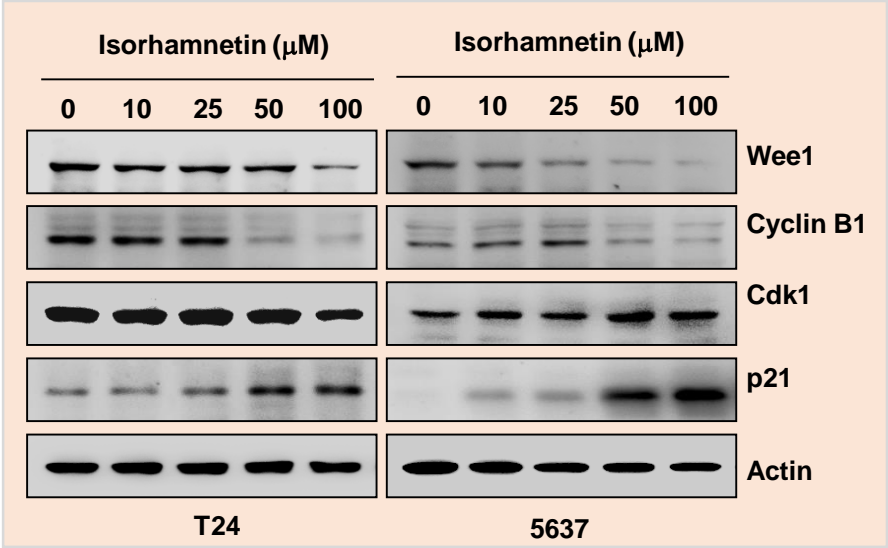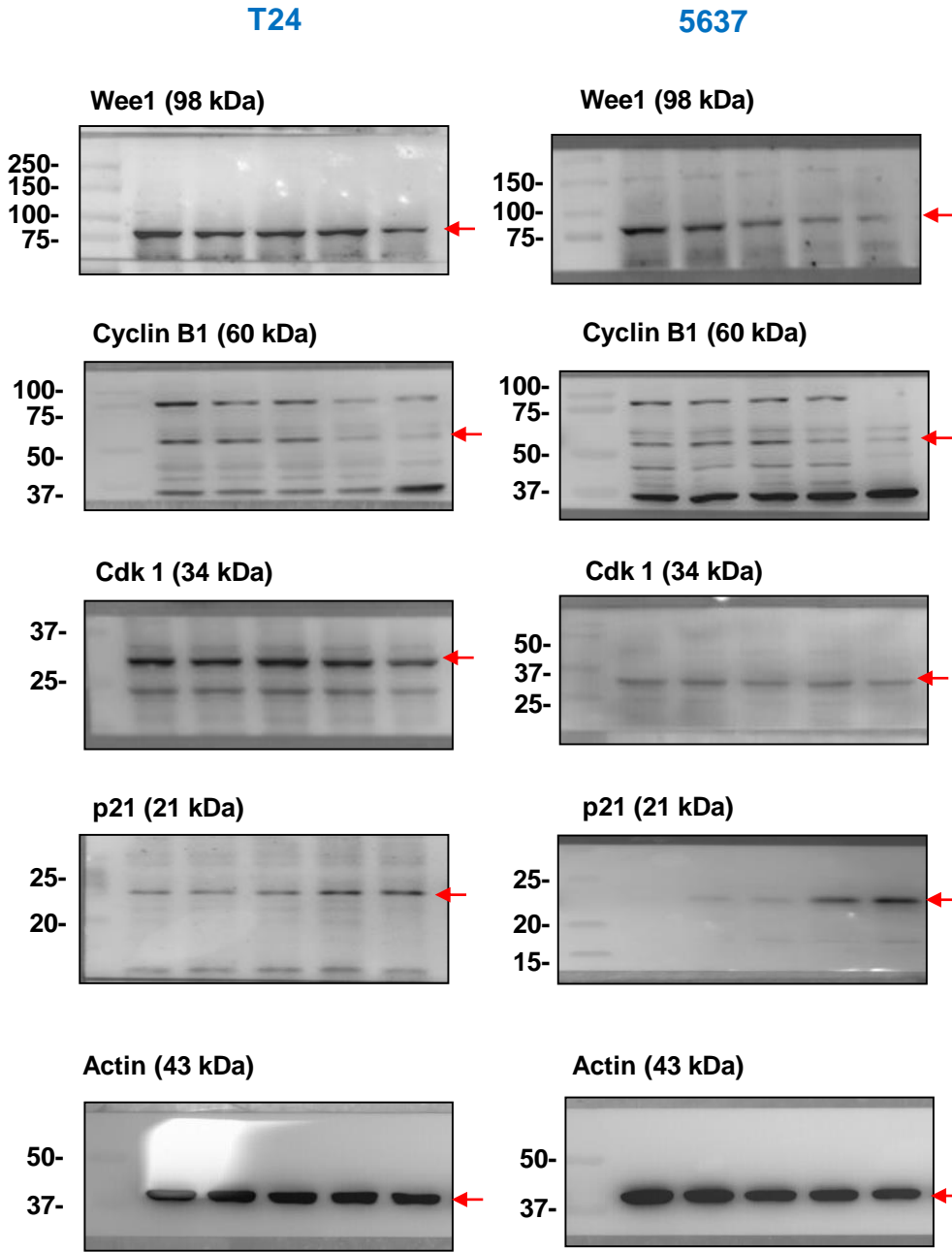

Figure 3-C

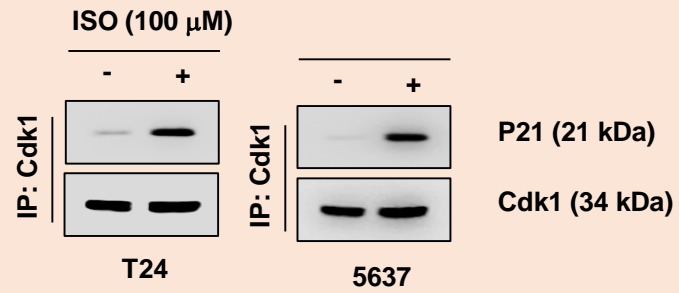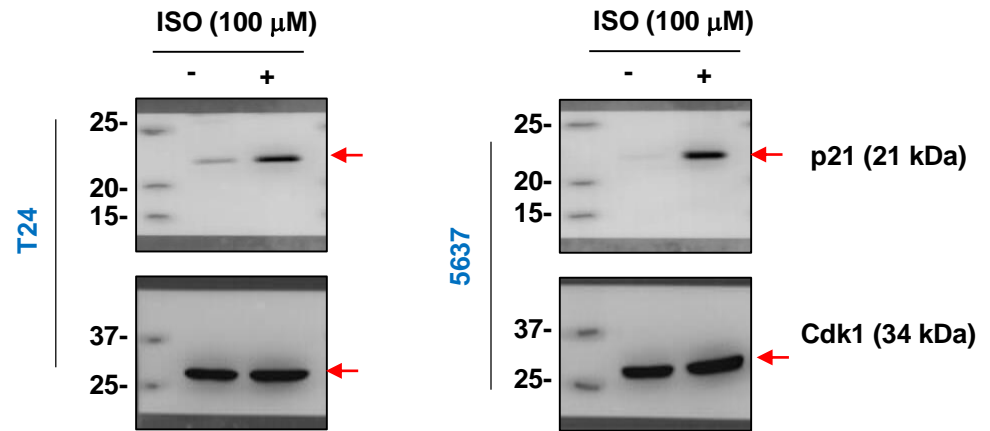

Figure 4-D : T24

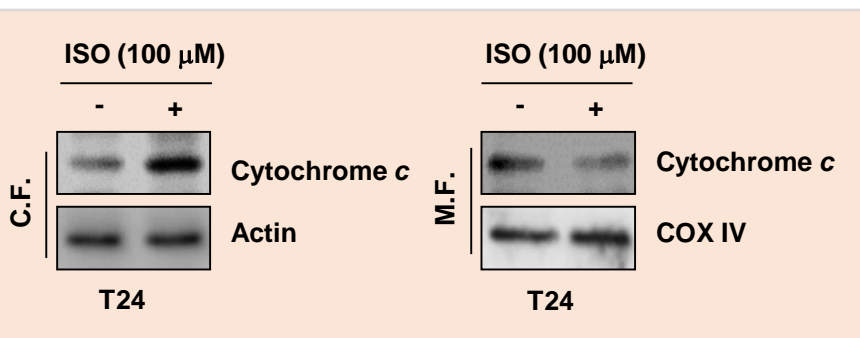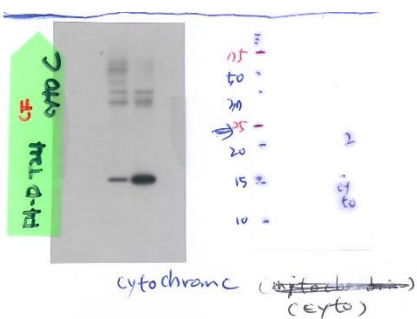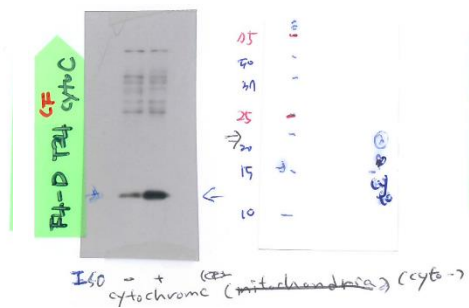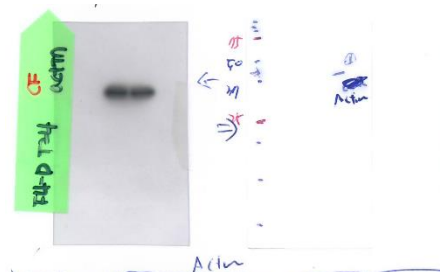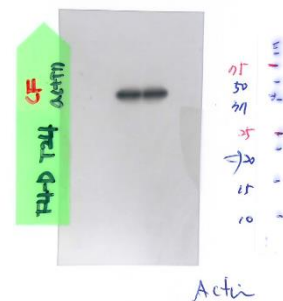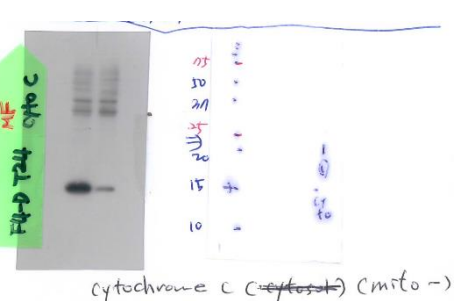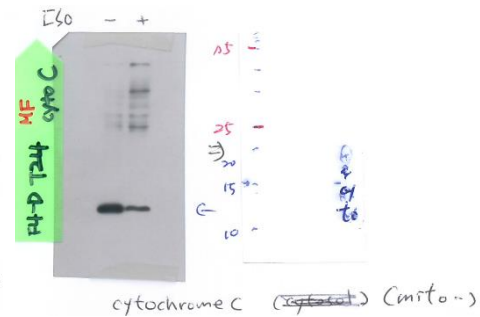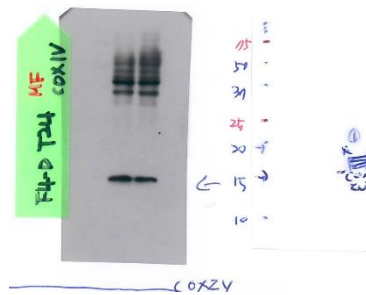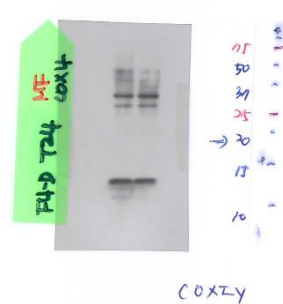

Figure 4-D : 5637

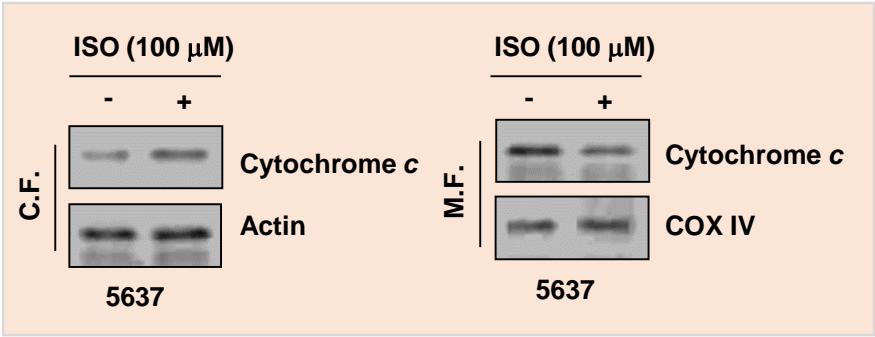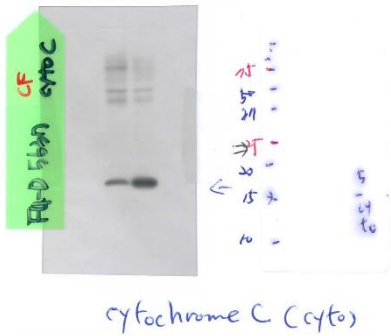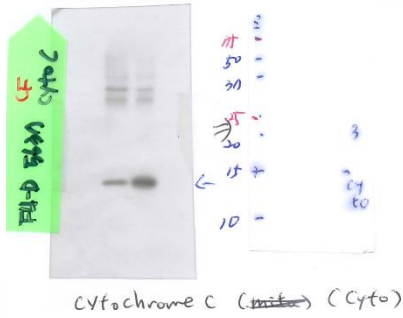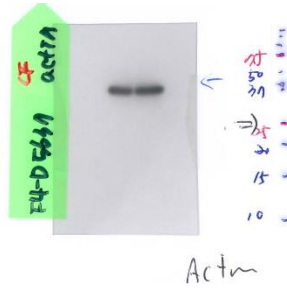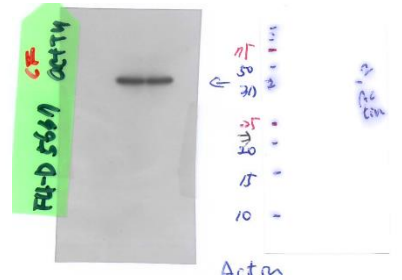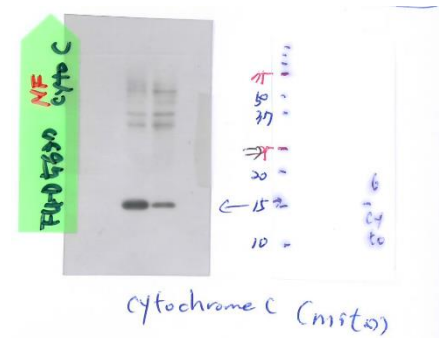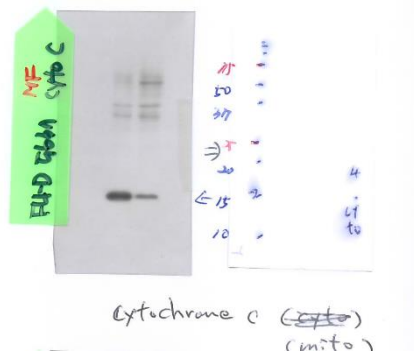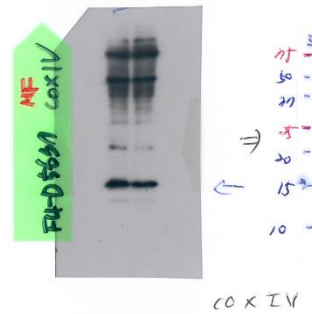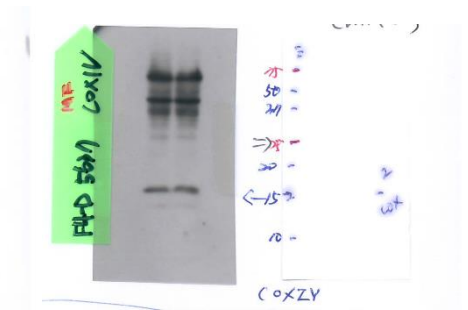

Supplement: Supplementary file 1 [file cancers-11-01494-s001.pdf]
